# Short-term anesthesia inhibits formalin-induced extracellular signal-regulated kinase (ERK) activation in the rostral anterior cingulate cortex but not in the spinal cord

**DOI:** 10.1186/s12990-015-0052-z

**Published:** 2015-08-14

**Authors:** Keri K Tochiki, Maria Maiarù, James R C Miller, Stephen P Hunt, Sandrine M Géranton

**Affiliations:** Department of Cell and Developmental Biology, University College London, London, UK

**Keywords:** Anesthesia, ERK, Anterior cingulate cortex, Spinal cord

## Abstract

**Background:**

The rostral anterior cingulate cortex (rACC) has been implicated in the negative affective response to injury, and importantly, it has been shown that activation of extracellular signal-regulated kinase (ERK) signaling in the rACC contributes to the full expression of the affective component of pain in rodents. In this study, we investigated whether administration of anesthesia at the time of injury could reduce phosphorylated-ERK (PERK) expression in the rACC, which might eliminate the negative affective component of noxious stimulation. Intraplantar hindpaw formalin stimulation, an aversive event in the awake animal, was given with or without general isoflurane anesthesia, and PERK expression was subsequently quantified in the rACC using immunohistochemistry. Furthermore, as numerous studies have demonstrated the importance of spinal ERK signaling in the regulation of nociceptive behaviour, we also examined PERK in the superficial dorsal horn of the spinal cord.

**Findings:**

Formalin injection with and without short-term (<10 min) general isoflurane anesthesia induced the same level of PERK expression in spinal cord laminae I–II. However, PERK expression was significantly inhibited across all laminae of the rACC in animals anesthetized during formalin injection. The effect of anesthesia was such that levels of PERK were the same in formalin and sham treated anesthesized animals.

**Conclusions:**

This study is the first to demonstrate that isoflurane anesthesia can inhibit formalin-induced PERK in the rACC and therefore might eliminate the unpleasantness of restraint associated with awake hindpaw injection.

## Findings

### Background

Pain processing involves neural activity generated by nociceptive stimuli in the spinal cord, the site of the initial key synapse of nociceptive primary afferents, and subsequently in brainstem and cortex. Importantly, the rostral anterior cingulate cortex (rACC) has been implicated in the negative affective response to noxious stimuli, as it has been shown that the rACC is required for noxious stimuli-induced associative learning in rodents [1]. Recent studies have also shown that stress induced activity in the ACC may explain how anxiety can potentiate chronic pain [2].

We hypothesised that general anesthesia at the time of injury would eliminate the affective component of noxious stimulation and thus should have an effect on subsequent molecular signaling in the rACC. We therefore examined phosphorylated-extracellular signal-regulated kinase (PERK) expression in the rACC following noxious formalin stimulation of the hind paw given with or without general isoflurane anesthesia. PERK is a widely used molecular marker of nociceptive stimulation. Phosphorylation of ERK in dorsal horn neurons occurs rapidly following noxious peripheral stimulation and is an essential signal leading to central sensitization [3, 4]. It has also been demonstrated that rapid activation of ERK in rACC regulates nociceptive-specific related negative affect, but not nociceptive behavioural responses per se [5]. Thus, the effect of general inhalation isoflurane anesthesia on PERK expression was examined in the spinal cord and the rACC.

### Results

#### There was no difference in formalin-induced PERK expression in the superficial dorsal horn with or without short-term anesthesia

**Fig. 1:**
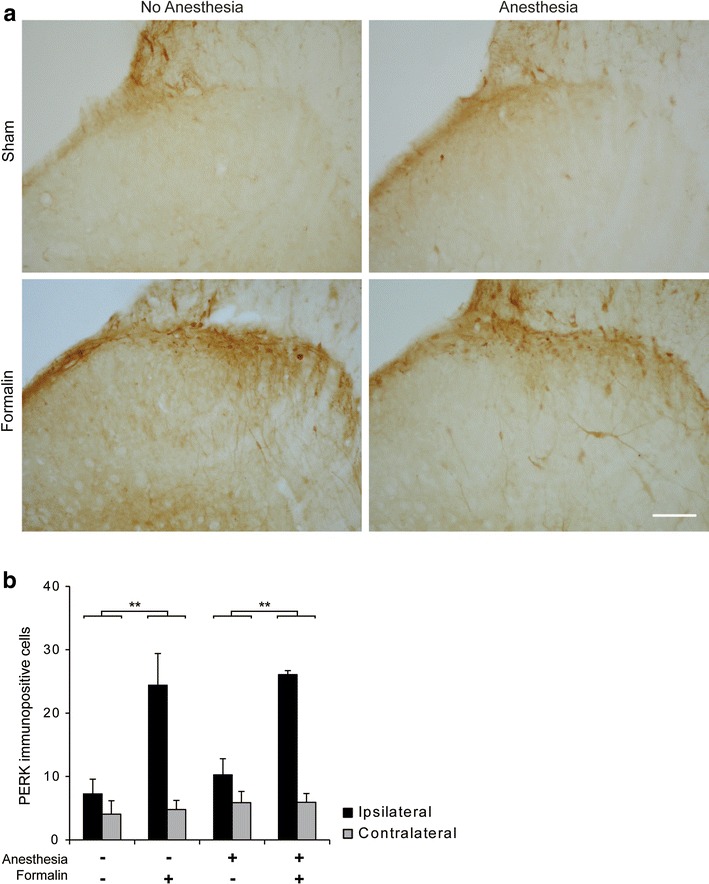
Short general anesthesia does not modulate formalin induced spinal PERK expression. **a** Typical PERK expression in the ipsilateral superficial dorsal horn for each treatment group. *Scale bar* 50 µm. **b** PERK counts. LSD post hoc, **P < 0.01, Formalin vs. Sham groups. Data presented as mean ± SEM (n = 3 each group).

Acute noxious formalin stimulation is known to induce ERK phosphorylation which persists up to 1 h post-injury in the spinal dorsal horn. PERK expression 30 min after formalin stimulation was significantly greater in formalin vs. sham treated animals (Fig. 1) (main effect of treatment; ANOVA F_(1, 16)_ = 23.4, p < 0.001). However, there was no difference in PERK activation between anesthetized and non-anesthetized animals (Fig. 1).

#### Anesthesia inhibited the expression of PERK in the rACC

**Fig. 2:**
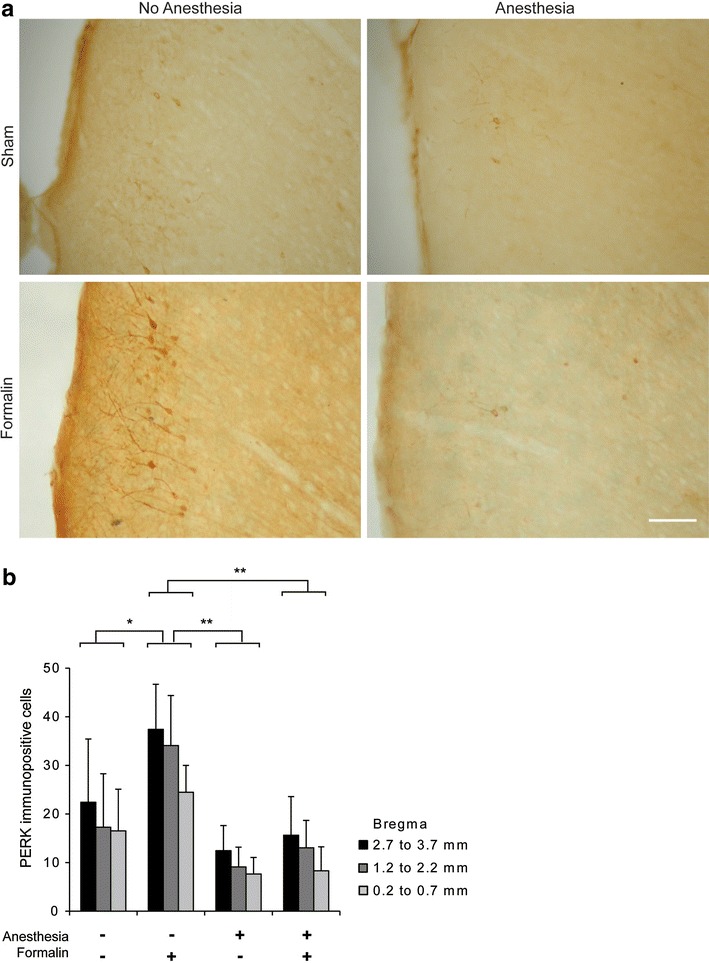
Short general anesthesia modulates formalin induced PERK expression in the rACC. **a** Typical PERK expression in area Cg1 of the rACC at Bregma 2.7 mm for each treatment group. *Scale bar* 50 µm. **b** Quantification of PERK expression across rACC Bregma points using bins. *P < 0.05, **P < 0.01, data presented as mean ± SEM (n = 3 each group).

We found a bilateral up-regulation of PERK across all laminae of the rACC 30 min post-formalin stimulation of the hindpaw, as reported by others [5, 6], and therefore combined the counts from the ipsilateral and contralateral side for further analysis. Strikingly, although anesthesia did not lead to differences in spinal cord PERK activation, it had a significant effect on PERK expression in the rACC (effect of anesthesia ANOVA F_(1,24)_ = 9.75, P < 0.01) (Fig. 2). LSD post hoc analysis revealed that PERK expression in the Form (+) and A (−) group was greater than in the Form (−) and A (−) group (P < 0.05), Form (−) and A (+) group (P < 0.01) and the Form (+) and A (+) group (P < 0.01). Interestingly, there was no difference between the Form (+) and A (+) group and the Form (−) and A (+) group suggesting that increased PERK expression in the rACC could only be seen with the combined condition of handling/restraint and formalin injection.

### Discussion

Experiments in this study aimed to identify whether anesthesia could modulate PERK activation in the dorsal horn and rACC caused by a dose of formalin widely used to induce acute hypersensitivity. Anesthesia had no effect on PERK expression in the superficial dorsal horn but inhibited PERK expression in the rACC.

#### General anesthesia and molecular signalling in the spinal cord

Here we report that short (<10 min) general anesthesia had no effect on PERK signalling in the superficial dorsal horn. While no study has directly investigated the effect of isoflurane anesthesia on nociceptive spinal PERK activity, isoflurane is thought to have an inhibitory effect on neural activity [7–10]. Moreover, studies have indicated that injectable anesthetics such as propofol, fentanyl, and pentobarbital [11–13] as well as inhalational anesthetics such as nitrous oxide and isoflurane [14] reduced c-Fos expression in the spinal cord. While isoflurane reduces c-Fos expression, it does not alter primary afferent peptide release into the spinal cord as investigated by internalisation of the NK1 receptor for SP in the dorsal horn [13]. Takasusuki et al. [13] suggested that while this result could implicate a role for non-peptidergic primary afferent activity accounting for the effects of general anesthesia, it is more likely that general anesthetics play a role in post-synaptic suppression of molecular nociceptive targets. PERK is more rapidly activated than c-Fos protein, and is suggested to be directly affected by NK1 receptor activation [15–17], suggesting that spinal PERK expression is unlikely to be modulated by anesthesia.

However, it is important to note that the depth and duration of the anesthesia are certainly critical factors. A previous study showed that PERK expression in the cerebral cortex was significantly inhibited by long (>10 min) compared to short (2–5 min) anesthesia [18]. In our experiments, animals receiving formalin with anesthesia were never subject to anesthetic for longer than 10 min.

#### General anesthesia reduces injury-induced molecular signalling in the rACC

Our results have shown that while ERK in the spinal cord was activated to the same degree following formalin injection in anesthetized and non-anesthetized animals, PERK activation in the rACC was strongly inhibited by anesthesia. These results suggest that the combination of the injection and the restraint is required to promote ERK signalling in the rACC. Due to the nature of the role of the rACC in nociceptive contextual affect, we propose that the reduction in rACC PERK expression in anesthetized animals is a direct result of the elimination of the aversive experience of live hindpaw injection.

The role of the rACC in the negative affective response to noxious stimuli was previously established by Johansen and Fields [1] and subsequent studies have demonstrated that inhibition of rACC signalling via pharmacological blockade of MAPK signalling or chemical lesions could eliminate the negative emotion associated with pain [5, 19, 20]. Our data therefore suggest that the absence of anesthesia promotes the negative affective component of the pain response associated with formalin injection. When inducing nociceptive signaling events in animal models, in particular when injecting the hindpaw, general anesthesia is often administered to minimize the unpleasantness of the injection procedure itself and the effects that this bear on the rACC signalling should be borne in mind. Finally, some evidence seems to support the idea that activity in the ACC following noxious stimulation is contextually dependent [21], and therefore reflect a function of sensory salience detection and not only nociceptive processing. Thus, our results may also support a functional role for the ACC in general arousal [21].

### Methods

#### Animals

All procedures were licensed under the Animals (Scientific Procedures) Act 1986. Experiments were performed using adult male Sprague–Dawley rats at 200–230 g at the beginning of the experiment and bred by the UCL Biological Services Unit. Animals were housed four maximum to a cage, with a 12 h light–dark cycle (lights on beginning at 08:00) and access to food and water provided ad libitum.

#### Experimental design and model of inflammation

There were four treatment groups (n = 3 each group):

1. Formalin injection-anesthetized: Form (+)–A (+)
2. Formalin injection- non-anesthetized: Form (+)–A (−)
3. Sham-anesthetized: Form (−)–A (+)
4. Sham- non-anesthetized (handling/restraint only): Form (−)–A (−)

Acute inflammation was induced using intraplantar hindpaw injection of 1 mg paraformaldehyde (33.3 µmol) in 20 µl for animals injected without anesthesia and 50 µl for anesthetized rats (due to Home Office licence limitations). Anesthesia never lasted more than 10 min. Rats without anesthesia were injected under light restraint in a glove (Form(+)-A(−)). Sham animal groups were either given general anesthesia only (Form(−)-A(+)) or light restraint only (Form(−)-A(−)) and none received injection.

Animals subject to anesthesia were returned to a recovery box and monitored until regaining consciousness. All animals were terminally anesthetized for perfusion 30 min following stimulation and transcardially perfused with heparinised saline (5,000 IU/l 0.9 % saline) followed by 4 % PFA/0.2 % NaF/0.1MPB. Spinal cords and brains were removed and post-fixed for 2.5–3 h in the same PFA solution and subsequently stored at 4 °C in 30 % sucrose/0.02 % azide/0.1 M PB until processing.

#### Immunohistochemistry

The L4–L6 region of the spinal cord and the rACC were isolated for sectioning on a freezing microtome. 40 µm serial coronal sections were taken and stored in 5 % sucrose/0.02 % azide/0.1 M PB until staining. PERK was detected using 3,3′-diaminobezidine (DAB) amplification. All staining of tissue was done free floating in volumes of 1 ml solutions with gentle rocking agitation. 3 × 10 min washes in 0.1 M PB were performed after incubation with all solutions except after incubation with blocking solution. Tissue sections were blocked for 1 h in 3 % normal goat serum (Vector, UK), 0.3 % triton X-100, and 2 % H_2_O_2_ in 0.1 M PB. The primary antibody PERK (1:400/TTBS brain, 1:500/TTBS cord; Cell Signaling) was added overnight (O/N) at room temperature in triton-tris-buffered saline (TTBS). The following day, a biotinylated secondary antibody (1:500/TTBS; Vector, UK) was subsequently applied for 2 h, followed by 1 h in ABC solution (1:1,000 of each Vectastain A and Vectastain B; Vector, UK). Sections were then developed using a DAB kit (Vector, UK). The reaction was terminated by transferring the sections into dH20 followed by 0.1 M PB (5 min each). Sections were mounted from 0.01 M PB and left to dry O/N before dehydration (dH20 followed by 2 × 1 min in each 70 % EtOH, 95 % EtOH, 100 % EtOH, Histoclear (National Diagnostics, UK). Slides were coverslipped directly from histoclear with DPX mountant (VWR, UK).

#### Imaging

Images were captured using a Nikon Coolpix E4500 digital camera attached to a Nikon Eclipse E800 microscope.

#### Quantification and analysis

Immunopositive cells for all sections were counted manually using a Leica DMR microscope in laminae I–II of the spinal cord and all laminae in cingulate area 1 (Cg1) of the rACC, defined by Paxinos and Watson in the Rat Brain Atlas [22] (Bregma 3.7 mm to 0.2 mm of the brain), with experimenter blind to treatment. Brain sections were assigned to one of the following coordinates (Bregma 3.7, 3.2, 2.7, 2.2, 1.7, 1.2, 0.7, 0.5 or 0.2) according to the plates described in the fourth edition of the Atlas of Paxinos and Watson. Sections were then divided into three areas (3.7–2.7 mm; 2.2–1.2 mm and 0.7–0.2 mm) and the four sections with the maximum response averaged per area. There were no significant differences between the ipsilateral and contralateral regions in Cg1 and therefore counts from both areas were combined. For spinal cord sections, the five sections within L4–L5 with the maximum response were used per animal and the average taken for the ipsilateral dorsal horn. All treatment group values were calculated as mean ± the standard error of each group mean (SEM). Statistical analyses were performed using IBM SPSS PC+ (Chicago IL, USA) or Microsoft Excel (Redmond WA, USA). Analyses were performed using 2-way analysis of variance (ANOVA) followed by relevant post hoc analysis. Data was checked for normal distribution using the Shapiro–Wilk analysis, and homogeneity of variance using Levene’s test prior to performing parametric analyses.

## Abbreviations

rACC: rostral anterior cingulate cortex
PERK: phospho-p44/42 MAPK (Thr202/Tyr204) extracellular signal-regulated kinase
i.pl.: intraplantar
